# Transcriptome Sequencing in the Preoptic Region of Rat Dams Reveals a Role of Androgen Receptor in the Control of Maternal Behavior

**DOI:** 10.3390/ijms22041517

**Published:** 2021-02-03

**Authors:** András H. Lékó, Rashmi Kumari, Fanni Dóra, Dávid Keller, Edina B. Udvari, Vivien Csikós, Éva Renner, Arpád Dobolyi

**Affiliations:** 1 Laboratory of Neuromorphology, Department of Anatomy, Histology and Embryology, Semmelweis University, H-1094 Budapest, Hungary; lekandras@gmail.com (A.H.L.); fanni.dora@gmail.com (F.D.); keller.david8@gmail.com (D.K.); 2 Department of Psychiatry and Psychotherapy, Semmelweis University, H-1094 Budapest, Hungary; 3 MTA-ELTE Laboratory of Molecular and Systems Neurobiology, Department of Physiology and Neurobiology, Institute of Biology, Eötvös Loránd University, H-1117 Budapest, Hungary; rashmi.kaul17@gmail.com (R.K.); edina328@gmail.com (E.B.U.); csikos.vivien@gmail.com (V.C.); 4 Human Brain Tissue Bank and Microdissection Laboratory, Semmelweis University, H-1094 Budapest, Hungary; evarenner@yahoo.com; 5 Department of Physiology and Neurobiology, Eötvös Loránd University, Pázmány Péter sétány 1C, H-1117 Budapest, Hungary

**Keywords:** preoptic area, hypothalamus, maternal behavior, postpartum mother, steroid receptor, rat dams, gene expression, RNA-Seq

## Abstract

(1) Background: Preoptic region of hypothalamus is responsible to control maternal behavior, which was hypothesized to be associated with gene expressional changes. (2) Methods: Transcriptome sequencing was first applied in the preoptic region of rat dams in comparison to a control group of mothers whose pups were taken away immediately after parturition and did not exhibit caring behavior 10 days later. (3) Results: Differentially expressed genes were found and validated by quantitative RT-PCR, among them NACHT and WD repeat domain containing 1 (Nwd1) is known to control androgen receptor (AR) protein levels. The distribution of Nwd1 mRNA and AR was similar in the preoptic area. Therefore, we focused on this steroid hormone receptor and found its reduced protein level in rat dams. To establish the function of AR in maternal behavior, its antagonist was administered intracerebroventricularly into mother rats and increased pup-directed behavior of the animals. (4) Conclusions: AR levels are suppressed in the preoptic area of mothers possibly mediated by altered Nwd1 expression in order to allow sustained high-level care for the pups. Thus, our study first implicated the AR in the control of maternal behaviors.

## 1. Introduction

Postpartum behavioral changes are critical parts of reproduction in mammals [1]. They may be accompanied with altered gene expression levels in brain areas responsible for their regulations. Rats represent a very good animal model to study mechanisms of postpartum changes in the brain. Females, which are not maternal, do not take care of pups or might actually attack the pups, while maternal females show caring behaviors, e.g., building of nest, retrieval of pups to the nest, suckling and reduced anxiety, as well as lactation [2]. Hormonal alterations, occurring in last days of pregnancy, play important part: level of estrogen increases, meanwhile progesterone decreases right before parturition. However, maternal behavior is maintained in the postpartum period, even if the level of reproductive steroid hormones becomes low due to lactational anestrous [2]. Thus, steroid hormonal effects contribute to the initiation, but are not required for maintenance of maternal behavior. Instead, inputs from the pups are critical for the maintenance of maternal motivation and behavior [3].

The medial preoptic area (MPOA) plays a critically important function in the control of caring behaviors. The number of activated neurons is markedly elevated in MPOA of caring females [4]. In addition, lesions of the MPOA prevent nest building and retrieving components of caring in females [5], while experimental activation of this area increases maternal responsiveness [6]. From a functional point of view, the ventral subdivision of the bed nucleus of stria terminalis (vBNST) located dorsal and lateral to the medial preoptic nucleus (MPN) is part of the MPOA as it plays a role in regulation of maternal behavior as the ventromedially located MPN and surrounding MPOA. The abovementioned hormonal effects on the caring behavior are also mediated by the MPOA. Injection of estradiol or prolactin directly to the MPOA causes fast appearance of maternal behavior [7,8]. Not only neuroendocrine inputs, but also pup stimuli, exert their action via the MPOA as somatosensory, olfactory, and auditory stimuli from pups are activating the neurons of MPOA [9,10]. The description of the alterations of gene expression in maternally behaving females in the MPOA is a possible way to understand which genes are responsible for the caring behavior of mothers. The preoptic area was investigated in previous microarray studies in this regard [11,12,13]. We previously performed a microarray study to compare preoptic mRNA levels 9 days after parturition between dams and mothers deprived of their pups immediately after parturition [11]. There were some genes which demonstrated alterations in their expression levels in lactating dams, which led to subsequent functional validation studies [14]. In another microarray study, preoptic gene expression was compared in postpartum females with or without pup-experience and in maternally sensitized or control virgins [13]. It was found that pup-exposure had significant positive effect on expression of genes related to dopamine and glucocorticoids, opioid and GABA-receptor genes; and negative effect for serotonin-related genes. In another study, lactating and virgin females were compared using high density oligonucleotide microarrays in the MPOA. Annotated genes containing autism spectrum disorder, bipolar disorder, and schizophrenia-related genes were identified [12].

The objective of the present study was to determine genes, which play a role in maternal adaptation in the preoptic area using RNA-sequencing (RNA-Seq) methods, which have not been used to study maternally altered gene expression. We examined gene expression 10 days following parturition in the MPOA of rat dams rearing their litter and pup-deprived mothers. The pups of the latter control group were removed immediately after parturition. The identification of proper control group is not obvious because lactating mothers have milk production in addition to behavioral changes as far as they raise and protect their offspring. Any experimental manipulation of the maternal group (e.g., taking away the litter for a limited time period or applying pharmacological cessation of milk production) may compromise their natural physiological and behavioral characteristics, therefore, we did not introduce any disturbance to the mothers. For controls, nulliparous females have often been chosen in previous experiments. However, we believe that mothers whose pups were taken away immediately after parturition represent a better control group because these animals went through all the hormonal and physiological changes of pregnancy. However, it must be acknowledged that these animals differ from mothers not only in maternal motivation and behavior but other differences are also present due to the absence of lactation in them. Dissection of preoptic area was performed on the 10th postpartum days because pup-deprived controls do not show maternal behavior by that time [15]. The levels of differentially expressed genes were confirmed by real-time RT-PCR and their distribution was described by in situ hybridization histochemistry (ISHH). A significantly altered gene is a regulator of androgen receptor (AR). Therefore, we used western blot to compare AR levels in lactating and pup-deprived mothers. In addition, we administered flutamide, an AR antagonist, into the lateral ventricle to examine the role of AR in maternal behavior and motivation.

## 2. Results

### 2.1. Pup-Deprived Mothers Showed Heavily Impaired Pup-Directed Behavior

**Figure 1:**
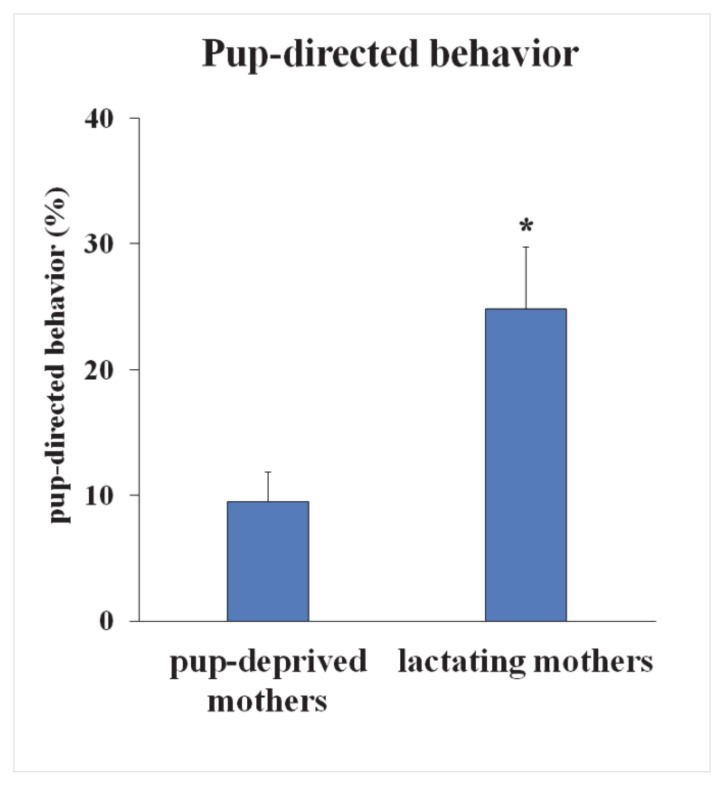
Pup-directed behavior of lactating and pup-deprived mothers. On the 10th day postpartum, maternal behavior of six lactating and six pup-deprived mothers was analyzed. Retrieval of pups, pup grooming, nest building, and sniffing of pups were considered as pup-directed behaviors. Pup-deprived mothers showed significantly decreased pup-directed behavior in the proportion of total time. (* = *p* < 0.05).

On the 10th day postpartum, maternal behavior of six lactating and six pup-deprived mothers was analyzed. Pup-deprived mothers showed significantly decreased pup-directed behavior assessed as its percentage of total time, which was 9.5 ± 2.4% compared to the lactating mothers 24.8 ± 4.9% (*p* < 0.05) (Figure 1). In addition, their pup-directed behavior consisted exclusively of sniffing, which is rather exploratory than specifically maternal behavior. In particular, animals whose pups were removed immediately after parturition did not retrieve pups at all.

### 2.2. Preoptic Transcriptome of Lactating and Pup-Deprived Mothers

In the project, we sequenced 12 samples on Illumina HiSeq Platform in total and generated an average of 76.41 ± 6.66 Mb total raw reads per sample (Table 1). The reads demonstrating inadequate-quality, adaptor-containing or unknown sequences, were not further considered in downstream analysis. The reads amount after filtering was 72.56 ± 6.42 Mb per sample. The percentage of clean reads was 94.83 ± 0.31%. On average, 85.93 ± 2.98% reads were mapped. The similarity of the mapping in all samples indicate the samples can be compared. 69.68 ± 2.43% of mapped reads aligned to a single unique location in the rat genome. The average gene mapping rate was 67.06 ± 2.4%. These alignment percentages suggest that all the sequenced data are comparable for the downstream analysis. 26,996 genes were identified, of which 25,999 were known genes. On average, 14,416 ± 131 genes showed expression (FPKM > 1) in lactating mothers, and 14,323 ± 148 genes in pup-deprived mothers. 1462 genes were expressed exclusively in lactating mothers; 793 in pup-deprived mothers; and 24,257 genes in both groups.

The amount of total raw reads, clean reads and their percentages are shown in preoptic samples of pup-deprived (D1–6) and lactating mothers (M1–6). Ratio of reads mapped to the reference genome, mapped reads aligned to a single unique location in the rat genome and rate of total gene mapping is seen in the table as well.

### 2.3. Differentially Expressed Genes in the MPOA of Lactating and Pup-Deprived Mothers

**Figure 2:**
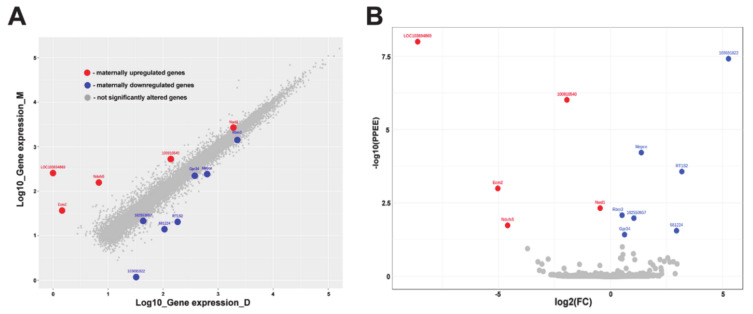
Representation of the expressional changes of the genes. (**A**) Scatter plot of the expressed genes in preoptic samples of mother (M) and pup-deprived controls (D). (**B**) Volcano plot shows the significance values and the fold change values to highlight the differentially expressed genes. Red dots represent maternally elevated, while blues represent maternally decreased differentially expressed genes. Genes without significant change are grey.

**Figure 3:**
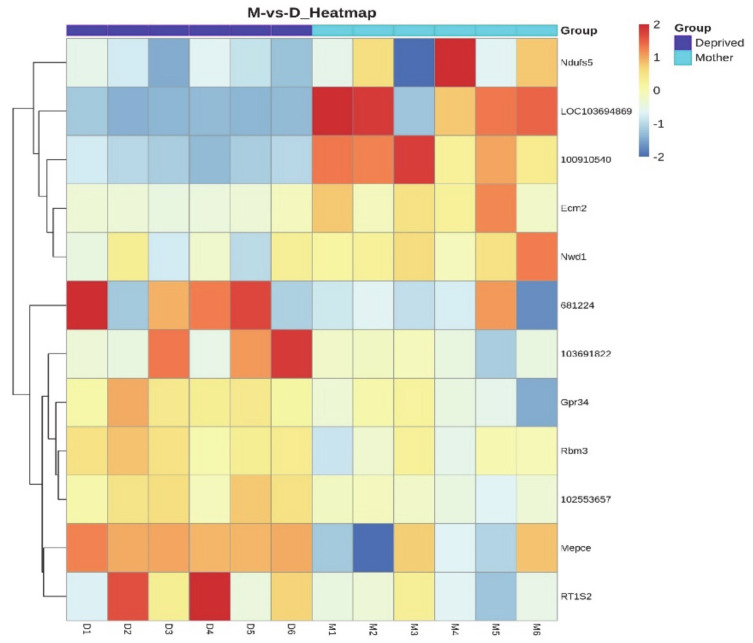
Differentially expressed genes in preoptic samples of mothers (M) and pup-deprived controls (D) are presented in a heatmap. The samples are shown horizontally, the DEGs are in Y axis. The color indicates the log10 transformed gene expression. Darkness means higher expression level.

After the quantification of genes in all samples, the differential expression was carried out by means of EBSeq between the two groups, mothers group and pup-deprived controls. There were 12 differentially expressed genes between the 2 groups. Among the 12 genes 5 genes were upregulated and 7 downregulated in maternal rats, as shown on scatter plot and heatmap (Figure 2 and Figure 3). In the heatmap, we can see that preoptic samples of lactating and pup-deprived mothers formed two different clusters. The list of 12 DEGs is shown in Table 2. From the 12 genes, eight were protein coding: Ndufs5 (coding NADH dehydrogenase (Ubiquinone) Fe-S protein 5, accessory subunit of mitochondrial respiration), Ecm2 (coding Extracellular matrix protein 2, involved in organization of extracellular matrix and collagen binding), LOC103694869 (coding Isochorismatase domain-containing protein 1, involved in metabolic process), and Nwd1 (coding NACHT and WD Repeat Domain Containing 1, controlling androgen receptor protein levels) showing elevated expression in lactating, Mepce (coding Methylphosphate Capping Enzyme protein, an RNA-binding methyltransferase), RT1-S2 (coding RT1 Class 1 S2 protein, involved in presenting foreign antigens to immune system), Rbm3 (coding RNA-binding protein 3, a translation factor), and Gpr34 (coding a G-protein coupled receptor 34) in pup-deprived mothers.

### 2.4. Validation of RNA-Seq

**Figure 4:**
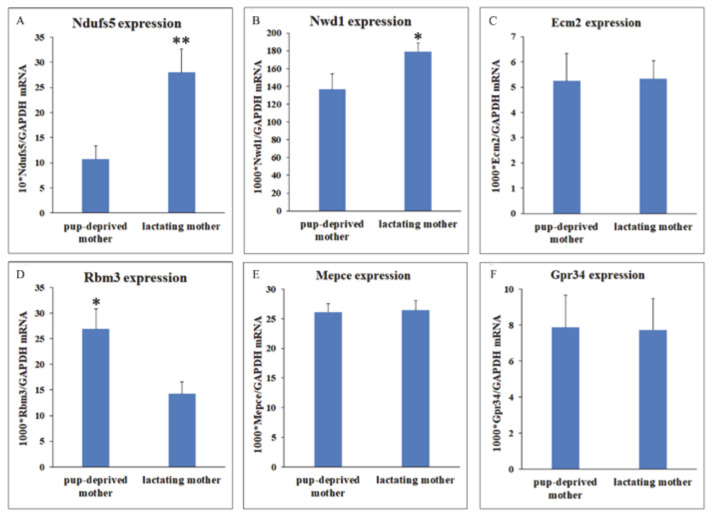
RT-PCR validation of DEGs in the MPOA of pup-deprived and lactating mothers (n = 11 for both groups). (**A**–**C**): mRNA levels of genes with higher expression in lactating mothers according to the RNA-Seq. Data, shown as ratio of GAPDH mRNA levels (mean ± SEM). Ndufs5 and Nwd1 mRNA levels were significantly higher in lactating mothers (** = *p* < 0.01, * = *p* < 0.05). Expression of Ecm2 did not differ significantly. (**D**–**F**): mRNA levels of genes with higher expression in pup-deprived mothers according to the RNA-Seq. Data, shown as ratio of GAPDH mRNA levels (mean ± SEM). Rbm3 mRNA level was significantly higher in pup-deprived mothers (* = *p* < 0.05). Mepce and Gpr34 expression did not differ significantly.

To verify changes in gene expression according to maternal adaptation, we performed qRT-PCR. We have chosen six functionally relevant genes from the DEGs: Ndufs5, Ecm2, Mepce, Nwd1, Rbm3, and Gpr34. According to the RNA-Seq results, we revealed significantly higher Ndufs5 mRNA levels in MPOA of maternal animals (represented as 10 * mRNA level/mRNA level of GAPDH): 27.98 ± 4.64 in maternal and 10.74 ± 2.66 in pup-deprived mothers (Figure 4A). We also succeed to validate the differential expression of Nwd1 (represented as 1000 * mRNA level/GAPDH): 179.04 ± 9.82 in lactating and 136.64 ± 17.62 in pup-deprived mothers (Figure 4B). In the RNA-Seq, Ecm2 showed elevated expression in lactating mothers, but we did not find a significant difference with qRT-PCR, as established by the mRNA levels in lactating and pup-deprived mothers respectively (1000 * mRNA level/mRNA level of GAPDH): 5.33 ± 0.71 vs. 5.24 ± 1.08 (Figure 4C). Between the genes with higher expression in pup-deprived mothers, we successfully validated the difference for Rbm3 (represented as 1000 * mRNA level/mRNA level of GAPDH): 14.24 ± 2.33 in mothers and 26.89 ± 3.94 in pup-deprived controls (Figure 4D). Expression of Mepce and Gpr34 did not show any significant difference with qRT-PCR (represented as 1000 * mRNA level/mRNA level of GAPDH): 26.4 ± 1.65 and 7.73 ± 1.75 in lactating mothers, 26.09 ± 1.41 and 7.87 ± 1.79 in pup-deprived mothers, respectively (Figure 4E–F).

### 2.5. Distribution of Differentially Expressed Genes in the Preoptic Area

**Figure 5:**
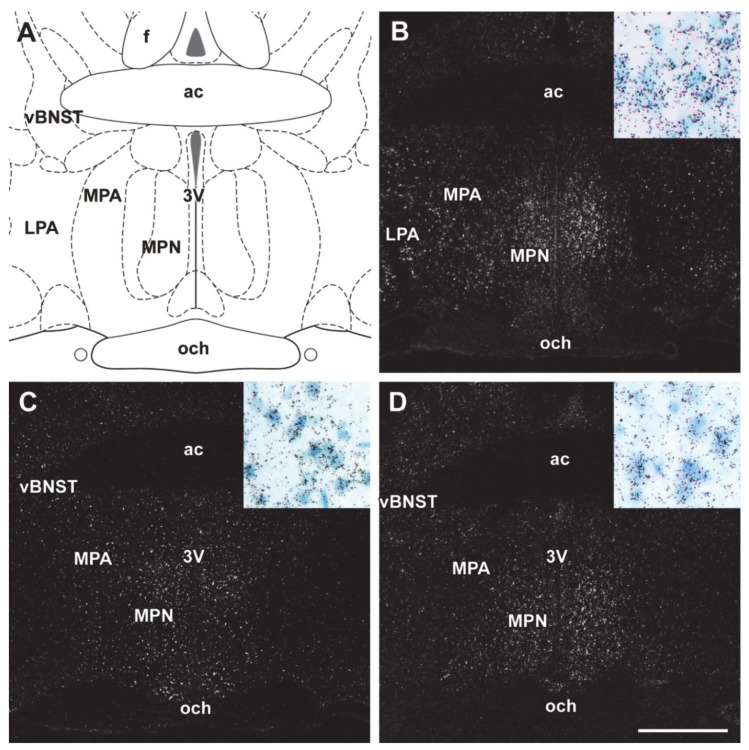
In situ hybridization histochemistry (ISHH) of DEGs in the preoptic area. White dots represent mRNA hybridization signal on the dark-field photomicrographs. Bright-field inlets demonstrate the autoradiography grains above labeled cells. (**A**) A drawing of the preoptic area in rat. (**B**) Ndufs5 mRNA hybridization signal was abundant in the MPN and also present in lateral preoptic area (LPA). (**C**) Nwd1 mRNA hybridization signal was seen in MPN, MPA, and also in the ventral subdivision of the bed nucleus of stria terminalis (vBNST). (**D**) Rbm3 mRNA expression was abundant in the MPN, MPA, vBNST. Scale bars =1 mm. Further abbreviations: ac—anterior commissure; och—optic chiasm; 3V—third ventricle.

In situ hybridization histochemistry was applied in lactating and pup-deprived mother rats, to localize the expression of Ndufs5, Nwd1 and Rbm3. In the case of Ndufs5, the hybridization signal was abundant in the whole MPOA, particularly in its medial part (Figure 5a). Nwd1 showed marked expression in the MPN, as well as BNST (Figure 5b). Hybridization signal of Rbm3 was notable in the MPOA, BNST, and lateral septum (LS) (Figure 5c).

### 2.6. Decreased Androgen Receptor (AR) Protein Levels in MPOA of Lactating Mothers

**Figure 6:**
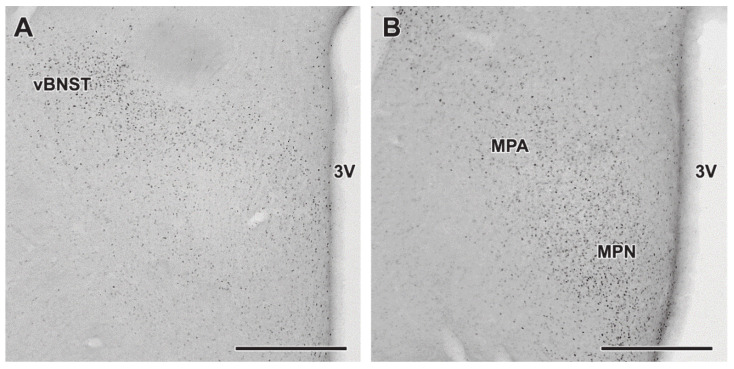
Distribution of Androgen Receptor (AR) detected by immunohistochemistry. Immunolabeling was performed in brains of mothers to detect AR in the preoptic region at 2 adjacent antero-posterior level (**A**,**B**). AR-immunoreactive cells (black dots) are abundant in MPN, MPA and vBNST. Scale bars = 500 µm. Further abbreviation: 3V—third ventricle.

**Figure 7:**
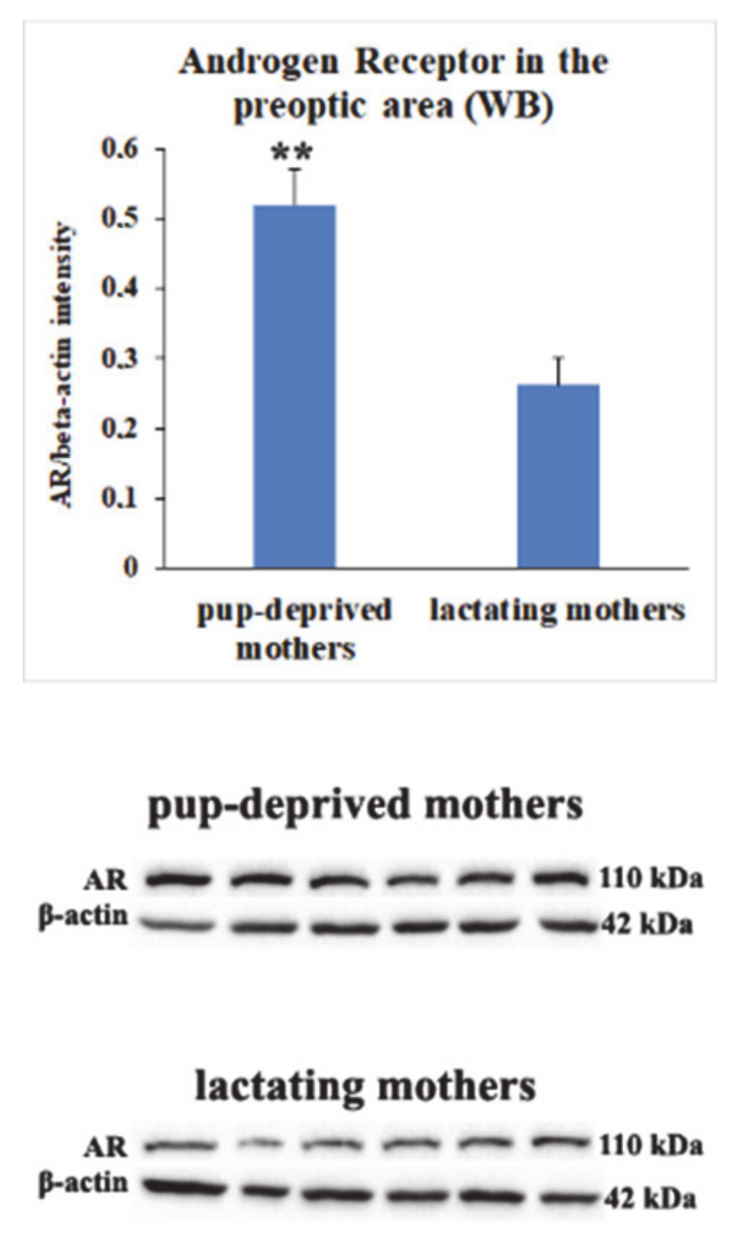
Androgen receptor (AR) protein levels in MPOA of pup-deprived and lactating mothers. Preoptic area was dissected from six pup-deprived and six lactating mothers on the 10th postpartum day. Data are expressed as β-actin intensity ration. Pictures of immunoblot bands are shown below. Western blot results show that AR protein levels are significantly higher in the MPOA of pup-deprived mothers. (** = *p* < 0.01).

Nwd1 was described before as a regulator of AR protein levels [16]. AR mRNA expression was not significantly different according to the sequencing results: expression in lactating mothers 141.80, expression in pup-deprived mothers 113.98, log2 fold change lactating/pup-deprived 0.313 and PPEE 0.999. With immunohistochemistry using AR antibody, we detected that AR is abundant in the MPOA, BNST and LS of lactating mothers (Figure 6), similar to Nwd1 expression as mentioned above. After this, we used western blotting to compare AR protein levels in MPOA of lactating and pup-deprived mothers (n = 6 − 6). Therefore, we dissected the preoptic area on the 10th postpartum day. We found that AR protein levels were decreased in lactating mothers (Figure 7). Results were as follows in the relation to b-actin intensity: 0.26 ± 0.04 in lactating and 0.52 ± 0.05 in pup-deprived mothers.

### 2.7. Intracerebroventricular (icv.) Administration of Androgen-Receptor Antagonist Flutamide Enhanced Pup-Directed Behavior

**Figure 8:**
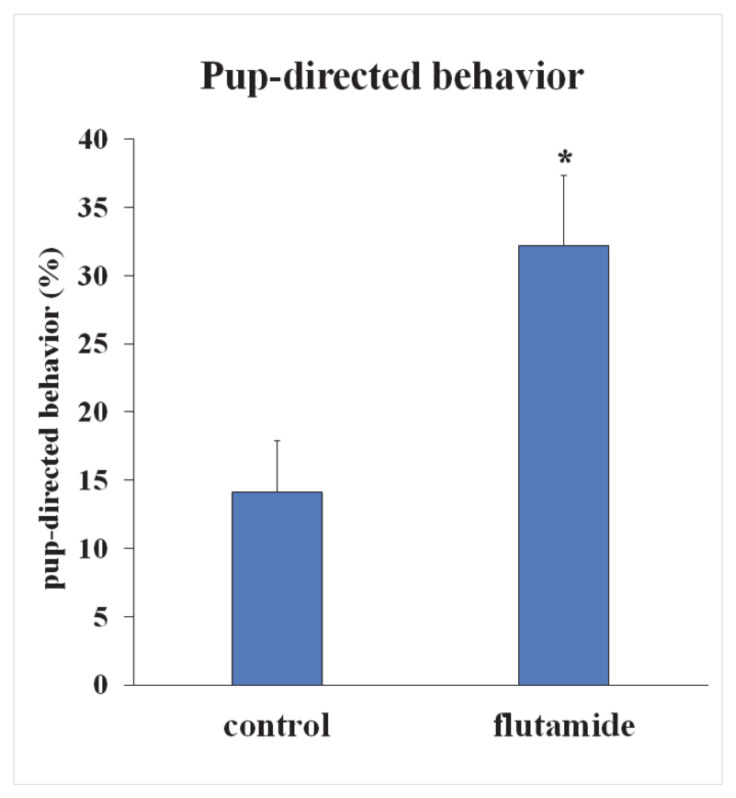
Pup-directed behavior of flutamide-treated and control mothers. Flutamide, a selective non-steroid antagonist of AR, was continuously administered icv. with a 0.5 mL/h speed via osmotic minipumps. One group of lactating mothers (n = 9) received flutamide and the control group (n = 9) received vehicle. On the sixth day postpartum, maternal behavior was analyzed. Retrieval of pups, pup grooming, nest building, and sniffing of pups were considered as pup-directed behaviors. Flutamide-treated mothers showed significantly increased pup-directed behavior shown as the ratio of total time. (* = *p* < 0.05).

To establish the role of AR in maternal behavior, flutamide, a non-steroidal selective antagonist of AR, was continuously administered icv. using osmotic minipumps implanted in dams on the second day after delivery. One group of lactating rats (n = 9) received flutamide dissolved in 75% ethanol and the control group (n = 9) received 75% ethanol. We analyzed maternal behavior on the sixth postpartum day as described previously: mothers were videotaped with three pups for 5 min after a 10 min long separation from their pups. Retrieval of pups, pup grooming and sniffing of pups were considered pup-directed behaviors. Flutamide-treated mothers showed significantly increased pup-directed behavior described in the proportion of total time, 32.21 ± 5.15% compared to 14.11 ± 3.78% in controls (Figure 8).

## 3. Discussion

### 3.1. Gene Expressional Changes in Mothers

RNA-Seq analysis has been used to describe gene expressional alterations in the preoptic area related to thermoregulation [17]. The present study demonstrates that the technique is suitable to describe gene expressional alterations related to the presence of pups in the postpartum period as well. An appropriately high quality of sequencing was performed based on the high number of total raw reads and the high total gene mapping ratio. Importantly, most of the genes in the preoptic area did not show alterations and had a symmetrical distribution. The examined samples separated well into 2 groups, maternal and pup-deprived, based on expressional data of the altered genes. Using strict criteria, only a relatively low number of genes demonstrated differentially expressed level between the 2 groups. It was expected as the same brain area was compared between similar animals. The animals were same age, same sex and conspecifics. Both groups underwent pregnancy and all related hormonal changes. However, the pup-deprived group did not show caring behavior towards foster pups in our test of parenting in accordance with literature data, which described reduced maternal behavior 7 days after removal of the pups at parturition, and no significant care on the 10th postpartum day [15]. Hence, the alteration in the differentially expressed genes likely occurred due to the difference in caring behavior. Consequently, the differentially expressed genes may have a role in maintaining maternal behavior.

### 3.2. DEGS in the MPOA of Lactating and Pup-Deprived Mothers

Quantitative RT-PCR was used in our study to validate RNA-Seq for individual genes. Since RNA-Seq is a systems biological technique whose analysis includes multiple hypothesis testing, positive and false negative changes are expected to occur. Indeed, we could not validate some of the DEGs. Altogether, the differential expression of three protein coding genes were validated: Ndufs5 and Nwd1 increased in maternal, while Rbm3 increased in pup-deprived animals.

#### 3.2.1. Ndufs5-NADH Dehydrogenase (Ubiquinone) Fe-S Protein 5

Ndufs5 encodes an iron-sulfur protein subunit of mitochondrial respiratory chain Complex I (NADH ubiquinone oxidoreductase) is member of complex of the oxidative phosphorylation. It takes electrons from NADH to ubiquinone. In the brain, mitochondria play a role in neuronal survival, and synaptic functioning. In neurons, mitochondria are highly dynamic in structure and function as they are carried to sites where ATP is necessary [18]. Dysfunction of Ndufs5 due to its SNP haplotype was associated with multiple sclerosis [19]. Mitochondrial operation is coupled to neuronal activity [20,21]. Therefore, significantly increased expression of Ndufs5 in MPOA of maternal dams may be a mark of increased mitochondrial function possible associated with pronounced activation of maternally involved neurons in the preoptic area.

#### 3.2.2. Nwd1-NACHT and WD Repeat Domain Containing Protein 1

NACHT and WD Repeat Domain Containing proteins belong to immunity proteins [22]. Originally discovered in fish, Nwd1 had orthologs in rodents and human, too [23]. In the adult brain, Nwd1 is prominently expressed in the hippocampus, the limbic system, the piriform and entorhinal cortices, the hypothalamus, the amygdala, the medial habenula, the red nucleus, and the substantia nigra [24]. As to its functions, recent evidence demonstrated that Nwd1 controls androgen receptor (AR) level as well as the activity of its signal transduction [16]. Furthermore, it was also suggested to regulate assembly of purine-synthesizing multienzyme complex called purinosome. Purinosome is necessary for axon and neurite outgrowth [25] and regulating excitatory signal transmission involving NMDA receptors. A reduced activity of Nwd1 decreases GluN2B level and also the phosphorylation in GluN2B subunit [26].

#### 3.2.3. Rbm3-RNA-Binding Motif Protein-3

Rbm3 is a positive regulator of translation as it promotes phosphorylation of translation initiation factors and supports polysome formation. Rbm3 belongs to cold-inducible, glycine-rich mRNA binding proteins consisting of a single highly conservative RNA recognition domain. They were suggested to have a role as mRNA chaperone-like proteins, which contribute to the maintenance of translation in stress. Rbm3 is a neuronal member of this family, which has significant expression level in the central nervous system. It is most abundant in the olfactory bulb, cortex, hippocampus, hypothalamus, cerebellum, tectum, pons, and the medulla. Rbm3 immunoreactivity is more abundant in neurons than in glial cell. In neurons, it is present in the synaptodendritic compartment. Rbm3 is induced in hypothermic conditions and in response to glutamatergic NMDA receptor activation [27]. Rbm3 is a key player in neuroprotective effect of hypothermia. In mouse models, the ability to regenerate synapses following cold stress was reduced when Rbm3 induction also decreased. Rbm3 over-expression resulted in sustained synaptic protection in mouse models of neurodegenerative disease, eliminating behavioral deficits and cell degradation, thereby increasing the survival time. On the other hand, knockdown of Rbm3 worsened synaptic elimination in neurodegenerative disorders and made disease symptoms worse, meanwhile inhibiting positive actions of cooling [28]. Rbm3 expression was significantly elevated in pup-deprived mothers in this work and also in the MPOA of dams in our previous microarray experiment [11]. Its reduced expression in mothers may be connected with the elevated core body temperature characteristic of lactation [29].

### 3.3. Distribution of DEGS with the Preoptic Area

The distribution of the three validated genes was revealed by in situ hybridization histochemistry, which also confirmed their appearance in the preoptic area. Ndufs5 showed dense appearance in the medial preoptic nucleus (MPN). Its distribution was similar to galanin-expressing neurons, but not to the preoptic neurons expressing c-fos as a sign of activation in response to pups. Localization of Ndufs5 cells overlapped with sex-steroid receptor and aromatase enzyme expressing neuronal clusters [30]. These neurons are either excitatory or inhibitory, expressing ERα and AR [31]. From this overlap of hormone-sensitive and Ndufs5-expressing neurons, it can be concluded that enhanced expression of Ndufs5 is a marker of increased responsiveness of preoptic area to hormonal stimuli. Nwd1 expression was abundant not only in the MPN but also in the anterolateral and anteroventral subdivisions of BNST. Its localization in the MPOA was very similar to the sexually dimorphic nucleus (SDN) and also overlapped with the sex-steroid receptor expressing neurons, mentioned above. The Nwd1-expressing cells in the BNST had a very similar pattern to parenting-activated galanin neurons in the anteroventral subdivision, and also to neurotensin-, proencephalin-, and maternally responsive CRH-expressing inhibitory neurons in the anterolateral subdivision [30]. Rbm3 expression was notable in the ventral subdivision of lateral septum in addition to the above mentioned MPOA.

### 3.4. Androgen Receptor (AR) as a Possible Modulator of Central Maternal Adaptation

Nwd1 is known as a regulator of AR protein levels [16]. Based on in vitro data using cell lines, downregulation of NWD1 reduces AR levels. The situation is, however, more complex in the brain where a variety of different cell types may express both NWD1 and AR. Although their distribution was similar in the preoptic area it is certainly likely that different cell populations may also possess NWD1 and AR. These cells could be excitatory and inhibitory neurons as well as glial cells. In some of these cells, desensitization mechanisms may also play a part as far as the interaction of NWD1 and AR. Therefore, the complex situations and mutual interconnections may lead to an average reduced AR levels in the preoptic area of mother rats. Therefore, we investigated the role of AR in the maternal adaptation of the central nervous system. The response to androgens is carried out by the well-established intracellular AR, a ligand-regulated transcription factor [32]. Upon ligand binding, the receptor gets into the nucleus thereby altering the expression of target genes [33]. AR-immunoreactive neurons are densely located in areas responsible for maternal responsiveness including MPOA. Its level is higher in males but its presence is also relatively high in females [33]. Here, we demonstrated that AR protein is present in the MPOA in mother rats, too. AR-immunoreactivity was abundant in the MPOA, especially in the MPN, but also in the vBNST. In the preoptic area, the localization of AR resembled to that of the expression of its modulator, Nwd1. AR-expressing neurons in the MPOA are mainly inhibitory GABAergic neurons, which play important role in the effects of androgens on corticotroph and gonadotroph axis, and also form neuronal clusters activated by parenting in mice mothers, fathers, and virgin females [30]. The effect of AR-expressing neurons of the MPOA exerts their actions on the corticotrophs by projecting to the paraventricular nucleus (PVN) [34]. It was also established that the non-steroidal selective antagonist of AR, flutamide, and testosterone in the MPN increase and decrease, respectively, the stress-evoked adrenocorticotropin hormone (ACTH) in the serum [35]. Similarly, anti-androgen microimplants increased luteinizing hormone (LH) secretion [36]. In this action, different preoptic cell types may participate in addition to GABAergic neurons, such as glutamatergic and peptidergic cells [37]. The density of AR-positive cells their AR immunoreactivity content changes during the estrous cycle. Estrous can be characterized with low AR level while during diestrous and metestrous, AR level was found to be elevated [33]. Alterations of AR protein level during the postpartum period have not been studied previously. Thus, we firstly established that AR is decreased in mothers while taking care of offspring. 

To learn the role of AR during parenting, we administered flutamide, a non-steroid selective AR-antagonist, to lactating mothers. Prolonged icv. administration resulted in a significantly increased pup-directed behavior. Flutamide is a selective, competitive antagonist of the AR without any effect on the progesterone, estrogen, glucocorticoid, and mineralocorticoid receptors [38]. Flutamide injections would be possible at different sites in the body. If peripheral application had been performed, non-neuronal actions would have made the conclusion difficult. A direct preoptic injection of flutamide would have been technically difficult as damages would have been made in the preoptic area, which are known to inhibit maternal behavior. Similar considerations lead us to avoid injection into the third ventricle. Instead, we injected flutamide into the lateral ventricle, which is big enough to safely accommodate the tip of the canul. The lateral ventricle is in the vicinity of the preoptic area. Therefore, it is reasonable to assume that flutamide can reach it by diffusion either directly or via the third ventricle. The disadvantage of intracerebroventricular as opposed to preoptic injection is that flutamide could potentially exert its action on the behavior though sites other than the preoptic area. However, the distribution of AR is limited within the brain, and it is reasonable to assume that the effect of flutamide on maternal behavior took place by a preoptic site of actions.

The most important androgens in females are dehydroepiandrosterone, dehydroepiandrosterone sulphate, androstenedione, testosterone, and dihydrotestosterone (DHT). The level of androgens in females generally amounts to only about 10–30% of the male values, which is, however, high enough to activate androgen receptor [37]. Actions of testosterone are exerted by direct binding to intracellular AR, via conversion to DHT by 5α-reductase and by DHT binding to AR, and also by conversion to estrogen by the aromatase, after which estrogen activates the estrogen receptor [37]. Investigation of mice lacking functional aromatase enzyme—as well as visualization of the activity of AR—suggest that, in contrast to previous views, the action of androgens is significantly exerted by AR [39,40]. Some previous studies suggested that circulating androgens control ovulatory cyclicity. Testosterone is necessary for the action of GnRH on LH-beta expression [41]. Thus, plasma testosterone and DHT levels in rats are highest at proestrus, fall to significantly lower levels at estrus and rise at metestrus and diestrus [42]. Alterations of hypothalamic testosterone levels show the same pattern [33]. In mice, testosterone has two peaks during pregnancy: mid-gestation and late-pregnancy [43]. Androgen levels during lactation have not been studied previously, but based on human studies, testosterone levels are reduced in mothers [44]. Androgens in females and women play other important roles and their disturbances can cause behavioral and physiological changes. In rodents, continuous week-long DHT treatment evoked a reduction in locomotor activity and also the duration in the open arms in the elevated plus maze, as an indicator of anxiety-like behavior [45]. High androgen level is also a characteristic of polycystic ovarian syndrome. In the postpartum and peripartum period, androgens correlated in humans with mood disorders. Androgen levels in venous blood samples collected from mothers after childbirth correlated positively with severity of depressive symptoms [46]. Markedly elevated testosterone levels were measured in postpartum depression at 24–28 h after childbirth, when compared to controls. High testosterone levels predicted postpartum depression [47]. In addition, correlation was found between elevated postpartum dehydroepiandrosterone sulphate levels and some psychiatric symptoms, such as anxiety [48].

## 4. Materials and Methods 

### 4.1. Animals

All animal experimentations were approved by the Animal Examination Ethical Council of the Animal Protection Advisory Board at the Eötvös Loránd University (PEI/001/37-4/2015). A combined 80 female Wistar rats were used. All rats were supplied with standard food and water. Anesthesia of animals was performed with a mixture, which was made of 66 mg/kg weigh ketamine and 14 mg/kg body weight xylazine.

### 4.2. Analysis of Maternal Behavior

Maternal behavior of six lactating and six pup-deprived mothers was analyzed on the 10th day postpartum. In another experiment, the same analysis was carried out using nine lactating mothers treated with icv. flutamide through osmotic minipumps and their controls (nine lactating mothers) on the sixth day postpartum.

Pups were taken away from their mothers 10 min. Then, three pups were given back to the cage placed in opposite corners. Similarly, 3 pups were given to pup-deprived mothers as well. Mothers were videotaped for 5 min. The videotapes were analyzed using Solomon Coder behavioral analysis software (https://solomon.andraspeter.com*/*) described previously [49]. A variety of different behavioral elements, such as retrieval of pups, pup grooming, sniffing of pups, environment exploration, activity out of the nest were separated. Retrieval of pups, pup grooming and sniffing of pups were considered as pup-directed behaviors. Therefore, their combined ratio in the total time was used to assess the intensity of pup-directed behaviors.

### 4.3. Microdissection of Brain Tissue Samples

**Figure 9:**
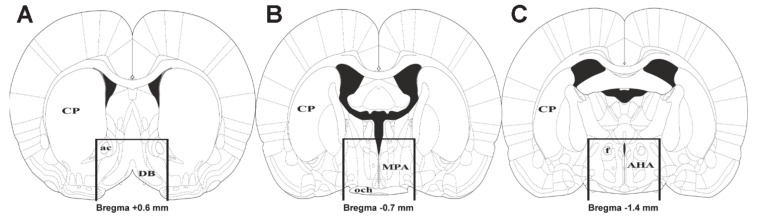
Position of the dissected tissue. The three drawings represent the level of the anterior cut (**A**), the middle level (**B**), and the posterior cut (**C**) in the coronal plane. Bars on the drawing represent vertical and horizontal cuts. Abbreviations: ac = anterior commissure, AHA = anterior hypothalamic area, CP = caudate putamen, DB = diagonal band of Broca, MPA = medial preoptic area, och = optic chiasm.

For RNA-Seq, brains of 6 primiparous lactating and six pup-deprived rats were used on the 11th day postpartum. For RT-PCR validation, we used additional 11 primiparous lactating and 11 pup-deprived mothers, their brains were also dissected on the 11th day postpartum. Immediately after the dissections of the brain, the preoptic area was microdissected as described by *Lékó* et al. [50] and shown as well (Figure 9).

### 4.4. RNA-Sequencing

RNA-Seq was performed from 12 brain tissue samples (six lactating and six pup-deprived preoptic sample) by BGI Technologies, Hong Kong on Illumina^®^ HiSeq Platfrom.

#### 4.4.1. Library Construction and Quality Check

The total RNA sample quality check was done by Agilent 2100 Bioanalyzer. The mRNAs were purified by oligo(dT) method and fragmented under certain conditions. Then first and second strand cDNA were produced. The cDNA fragments were end reparated. After that, they were surrounded by adapters and appropriate size fragments were PCR amplified.

#### 4.4.2. Alignment and Quantification

Firstly, insufficient quality reads were eliminated with SOAPnuke software (BGI Technologies, Hong Kong, China, version v1.5.2). The remaining reads were then mapped to the reference genome by means of HISAT2 (Hierarchical Indexing for Spliced Alignment of Transcripts; Center for Computational Biology, Johns Hopkins University, version v2.0.4). After genome mapping, StringTie (Center for Computational Biology, Johns Hopkins University, version v1.0.4) was applied for the reconstruction of transcripts. Then, novel transcripts were determined with Cuffcompare (a tool of Cufflinks^®^, software version v2.2.1). The potential of these transcripts to code mRNA was examined with CPC (Peking University, software version v0.9-r2). Subsequently, we mapped clean reads to reference with Bowtie2 (Bowtie^®^, software version v2.2.5) and calculated gene expression with RSEM (University of Wisconsin-Madison, version v1.2.12). Ebseq method was used to identify the DEGs between the two groups. EBSeq is based on empirical Bayesian model [51]. The >0.4 and <0.4 log2fold change was set along with <0.05 PPEE (Posterior probabilities of being equivalent expression) for differentially expressed genes as upregulated and downregulated genes, respectively.

### 4.5. Quantitiative RT-PCR

RT-PCR was performed using SupersciptII (Invitrogen) as described before [50]. The applied primers were: CCTTACAAGAATGCCGCTCG and TCTCCAGCGGAAAACAGAGC for Ndufs 5, TGTCATCACAACAGGGAGCC and CATGGGGCAGTGTCTTGACT for Nwd1, AGAGCTATGTCGCCCTATGC and GGGGACTTGAGGCATGGATC for Ecm2, GCTGACTGGCCAATGATCCT and AGACACTGCAAACCACTCCC for LOC103694869, TTGTTGTCAAGGACCGGGAG and TCTCTAGACCGCCCATACCC for Rbm3, CTTGGATGGAGCGGACACAT and AGGAGCTGAACTGTTCTGGC for Mepce, AGGGCAATAACCACCAAGCA and GGGCTGTCGTGGCATATTTG for Gpr34 and TGCCACTCAGAAGACTGTGG and GTCCTCAGTGTAGCCCAGGA for GAPDH.

### 4.6. Production of Hybridization Probes for Ndufs5, Nwd1, and Rbm3

Hybridization probes developed as previously described [52], although with some modifications. As described above, total RNA was isolated from frozen MPOA. Total RNA level was adjusted to 2 µg/µL for each sample. Then, cDNA was synthesized and amplified with iTaq polymerase (BioRad, Hercules, CA, USA). The primers were the same as above for RT-PCR. The PCR products were purified from gel and applied as templates in PCR reactions, using the same primers specific for Ndufs5, Nwd1, and Rbm3 except the reverse primers were supplemented with T7 RNA polymerase recognition site. PCR products were purified from gel again and used as templates in subsequent PCR reactions with the same forward and T7-containing reverse primers. Products of these reactions were purified and used to synthesize labelled RNA probes for in situ hybridization. 

### 4.7. In Situ Hybridization Histochemistry

To determine the distribution of the expression of validated DEGs, in situ hybridization histochemistry was performed as described before [11]. Briefly, 12 µm thick sections were cut from brains of two lactating mothers. Antisense [35S] UTP-containing probes were produced with T7 RNA polymerase (Ambion, Austin, TX, USA) from the above-described DNA probes specific for Ndufs5, Nwd1, and Rbm3.

### 4.8. Androgen Receptor (AR) Immunohistochemistry

Immunohistochemistry was performed as previously [53]. Briefly, two lactating rat mothers were anesthetized and transcardially perfused. Brain sections were prepared, on which, anti-Androgen receptor antibody (Sigma Aldrich, cat. no. 06–680) was used at a ratio of 1:500.

### 4.9. Western Blot Analysis

The preoptic areas of the hypothalamus from six lactating and six pup-deprived rat mothers were obtained for western blotting. Purification of proteins was carried out by radioimmunoprecipitation assay (RIPA) buffer. Then, the samples were centrifuged at 12,000× *g* for 30 min at 4 °C. Protein measurement was performed with BCA kit (Sigma-Aldrich, cat. no. BCA1-1KT). Proteins (25 μg per lane) were separated by standard SDS-PAGE and transferred to nitrocellulose (Bio-Rad, Cat. No. 1620112). Anti-Androgen receptor antibody (Sigma Aldrich, Cat. No. 06–680) was applied at a ratio 1:1000 and incubated at 4 °C for a day. The bound antibodies were labeled with anti-rabbit (1:2000; Jackson ImmunoResearch, Cat. No. 711035152) horseradish peroxidase-conjugated secondary antibody followed by visualization with Gel Doc XR+ imaging system (BioRad) using Clarity Western ECL Substrate (BioRad Laboratories, Cat. No. 170–5060). The molecular weight of the labeled proteins were measures with PageRuler Prestained Protein Ladder (cat. no. 26616, Thermo Scientific, Waltham, MA, USA).

### 4.10. Implantation of Intracerebroventricular Cannulae

Implantation of intracerebroventricular cannulae was performed as described previously [50]. Two days after delivery, rats were separated into two groups to determine the role of Androgen receptor in maternal behavior: icv. infusion of flutamide, a non-steroidal selective antagonist of AR (n = 9) and control group (n = 9). Osmotic minipumps administering the substance for 2 weeks (ALZET Micro-Osmotic Pump Model 2002, Durect™) were used to inject flutamide (Sigma, St. Louis, MO, USA; 25 µg/µL, in 75% ethanol; 12 µg/h), or 75% ethanol.

## 5. Conclusions

Despite all the above-described physiological actions and pathophysiological roles of androgens in females, their role in maternal behavior has not been investigated. Thus, our finding is novel that AR antagonism strengthens maternal responsiveness in lactating rat mothers, and suggests that maternal behavior is inhibited via the AR. This conclusion supports previous studies suggesting that androgens promote male and suppress female sexual behaviors not only by their organizational but also by their activational actions [37]. Furthermore, our finding is also in concordance with human data describing positive correlation between postpartum mood disorders and serum androgen levels.

## Figures and Tables

**Table 1:** Quantity and quality characteristics of the RNA-Seq

	Total Raw Reads (Mb)	Clean Reads (Mb)	Clean Reads Ratio (%)	Total Reads Mapping Ratio (%)	Uniquely Reads Mapping Ratio (%)	Total Gene Mapping Ratio (%)
D1	57.27	52.8	92.19	75.12	61.22	57.25
D2	58.08	54.54	93.9	74.14	60.16	58.19
D3	51.25	48.8	95.23	94.28	76.39	70.65
D4	60.63	57.1	94.17	93.51	76.47	72.67
D5	62.55	59.42	95	74.3	60.26	58.78
D6	61.46	58.47	95.14	74.43	60.17	57.58
M1	102.26	96.28	94.15	94.69	77.19	75.08
M2	90.68	87.18	96.14	94.52	76.76	74.15
M3	92.07	87.73	95.28	93.71	76.05	72.68
M4	120.24	114.73	95.42	94.58	76.41	75.51
M5	100.04	96.01	95.97	94.58	76.04	75.19
M6	60.36	57.6	95.42	73.27	59.06	56.96

**Table 2:** Differentially expressed genes in the preoptic area of lactating and pup-deprived mothers. The genes with elevated expression in lactating mothers are shown in red and those with elevated expression in pup-deprived mothers are blue. The genes were filtered as differentially expressed genes if their log2 Fold Change was between −0,4 and +0.4 and their PPEE (posterior probabilities of being equivalent expression) value were ≤0.05.

Gene ID	Unigen ID	Gene Symbol	Protein	Expression in Lactating Mothers	Expression in Pup-Deprived Mothers	Log2 (Lactating/Pup-Deprived)	PPEE	Function
103694869	Rn.1166.	LOC103694869	Isochorismatase domain-containing protein 1	254.936	0,000	8.612	0	Metabolic process
100910978	Rn.106785.	Ecm2	Ecm2 (Extracellular matrix protein 2)	35.792	0.46	6.281	0.00102	Organization of extracellular matrix, collagen binding
100363268	Rn.156413.	Ndufs5	NADH dehydrogenase (Ubiquinone) Fe-S protein 5	155.64	5.81	4.744	0.0186	Accessory subunit of the mitochondrial membrane respiratory chain
100910540	-	LOC100910540	-	525.556	135.613	1.954	0.000000966	-
100364350	-	Nwd1	NACHT And WD Repeat Domain Containing 1	2644.637	1919.119	0.463	0.0048	Control of androgen receptor (AR) protein steady-state levels.
114488	Rn.18057	Rbm3	RNA-binding protein 3	1530.588	2190.024	-0.517	0.00834	Regulation of translation, cold-inducible mRNA binding protein
554353	Rn.163444	Gpr34	G Protein-Coupled Receptor 34	235.54	364.333	-0.629	0.0384	G-protein coupled signal transduction
102553657	-	LOC102553657	-	20.299	42.477	-1.065	0.0105	-
304361	Rn.198943.	Mepce	Mepce protein (Methylphosphate Capping Enzyme)	243.175	631.98	-1.378	0.00006	RNA-binding methyltransferase
681224	-	LOC681224	-	13.044	105.041	-3.01	0.0281	-
24994	Rn.222446	RT1-S2	RT1 Class 1 S2	19.498	182.707	-3.228	0.0002	Involved in the presentation of foreign antigens to the immune system.
103691822	-	LOC103691822	-	0.178	31.412	-7.463	0.00000003	-

## Abbreviations

ac	anterior commissure
ACTH	adrenocorticotropin
CP	caudatus putamen
Cx	cerebral cortex
DAB	3,3-diaminobenzidine
f	fornix
FITC	fluorescein isothiocyanate
GAPDH	glyceraldehyde-3-phosphate-dehydrogenase
GnRH	gonadotropin releasing hormone
LH	gonadotropin releasing hormone
LS	lateral septal nucleus
LV	lateral ventricle
MPA	medial preoptic area
MPN	medial preoptic nucleus
och	optic chiasm
PB	phosphate buffer
PVN	paraventricular hypothalamic nucleus
vBNST	ventral subdivision of the bed nucleus of the stria terminalis
3V	third ventricle
